# Associations of the HER2DX Genomic Test with Biological and Pathologic Features in HER2-Positive Breast Cancer

**DOI:** 10.1158/1078-0432.CCR-25-3123

**Published:** 2025-12-01

**Authors:** Esther Sanfeliu, Anabel Martínez-Romero, Mercedes Marín-Aguilera, Sandra Cobo, Blanca González-Farré, Eva Hernandez-Illan, Pedro Jares, Joan Antón Puig-Butillé, Montserrat Muñoz, Raquel Gómez-Bravo, Marta Tapia, Cristina Tebar, Cristina Saura, Santiago Escrivà-de-Romaní, Jesús Soberino, Javier Cortés, Serafin Morales, Kepa Amillano, Laia Paré, Patricia Villagrasa, Wesley Buckingham, Francisco Pardo, Joel S. Parker, Fara Brasó-Maristany, Eva Ciruelos, Rodrigo Sánchez-Bayona, Olga Martinez-Sáez, Juan Miguel Cejalvo, Aleix Prat

**Affiliations:** 1 Department of Pathology, Biomedical Diagnosis Center, Hospital Clínic of Barcelona, Barcelona, Spain.; 2 Translational Genomics and Targeted Therapies in Solid Tumors, August Pi i Sunyer Biomedical Research Institute (IDIBAPS), Clinic Barcelona Comprehensive Cancer Center, Barcelona, Spain.; 3 Department of Medicine, University of Barcelona, Barcelona, Spain.; 4 Reveal Genomics, S.L., Barcelona, Spain.; 5 Molecular Biology Core, Hospital Clinic of Barcelona, Barcelona, Spain.; 6 Medical Oncology, Hospital Clinic of Barcelona, Barcelona, Spain.; 7 Medical Oncology, Hospital Clinic of Valencia, Valencia, Spain.; 8 Vall Hebron Institute of Oncology (VHIO), Barcelona, Spain.; 9 IOB-QuirónSalud, Barcelona, Spain.; 10 IBCC, Barcelona, Spain.; 11 Medical Oncology Department, Arnau de Vilanova, Lleida, Spain.; 12 Institut d’Oncologia de la Catalunya Sud (IOCS), Hospital Universitari Sant Joan de Reus, IISPV, Universitat Rovira i Virgili, Reus, Spain.; 13 Medical Oncology Department, Hospital Universitario 12 de Octubre, Instituto de Investigación Sanitaria Hospital 12 de Octubre (imas12), Madrid, Spain.

## Abstract

**Purpose:**

HER2DX is a validated genomic assay used to support treatment decisions in early-stage HER2-positive (HER2+) breast cancer. It provides three scores: relapse risk, likelihood of pathologic complete response (pCR), and *ERBB2* mRNA expression. This study aimed to evaluate the association between HER2DX and histopathologic features and assess its relationship with pCR after neoadjuvant therapy.

**Experimental Design:**

Patients with newly diagnosed stage I to III HER2+ breast cancer were analyzed based on available HER2DX results during routine care in Spain (January 2022–June 2025). Centralized HER2DX testing was performed on formalin-fixed, paraffin-embedded tumor samples. Histopathologic analysis included tumor grade, hormone receptor status, histologic subtype, Ki67 index, HER2 IHC score, stromal tumor-infiltrating lymphocytes (TIL), tertiary lymphoid structures, and spatial immune distribution. Univariate and multivariable logistic regression analyses were conducted to identify factors associated with pCR after neoadjuvant trastuzumab-based therapy.

**Results:**

A total of 410 HER2+ tumors were analyzed, and 250 patients received neoadjuvant trastuzumab-based therapy with available surgical outcomes (36% achieved a pCR). HER2DX pCR scores were significantly associated with all eight histopathologic features, whereas relapse risk and ERBB2 scores were associated with five and two, respectively. TIL correlated with the immune/immunoglobulin signature (r = 0.59), and Ki67 with the proliferation signature (r = 0.50). The HER2DX pCR score remained the only independent predictor of pCR in multivariable analysis (OR, 1.77; 95% confidence interval, 1.08–2.97; *P* = 0.030).

**Conclusions:**

HER2DX reflects key biological and pathologic features of HER2+ breast cancer and independently predicts pCR, supporting its utility for individualized treatment decision-making.

**Translational Relevance**

HER2DX is a clinically validated genomic assay that supports therapeutic decision-making in early-stage HER2-positive (HER2+) breast cancer by integrating three complementary readouts: risk of recurrence, likelihood of pathologic complete response (pCR), and *ERBB2* mRNA expression. In this multicenter study of 410 tumors tested in routine practice, HER2DX scores were associated with key histopathologic features, including tumor grade, hormone receptor status, histologic subtype, proliferation, immune infiltration, and HER2 protein expression. Among 250 patients treated with neoadjuvant trastuzumab-based therapy, the HER2DX pCR score emerged as the only independent predictor of pCR, reinforcing its clinical utility beyond standard pathology. Correlation analyses revealed that HER2DX captures luminal differentiation, proliferative activity, immune activation, and HER2 signaling—biological dimensions not fully reflected by conventional histopathologic evaluation. These findings provide independent biological and clinical validation of HER2DX and support its integration into routine workflows to personalize treatment selection in early-stage HER2+ breast cancer.

## Introduction

HER2-positive (HER2+) breast cancer represents a biologically heterogeneous subtype of breast cancer (1, 2), affecting 15% to 20% of patients and defined by overexpression or amplification of the *ERBB2* gene (3). Although HER2-targeted therapies have significantly improved patient outcomes (4, 5), there is increasing clinical need to optimize treatment strategies based on individual tumor biology. A primary aim in early-stage disease management is risk stratification to determine who may safely receive less-intensive therapy and who may benefit from treatment intensification.

HER2DX is a validated genomic test developed specifically for early-stage HER2+ breast cancer (6–9). It measures the expression of 27 genes grouped into four biological signatures—immune/immunoglobulin (IGG), luminal differentiation, proliferation, and HER2 amplicon expression—and produces three clinical scores: a relapse risk score, a pathologic complete response (pCR) likelihood score, and an *ERBB2* mRNA expression score. These scores have been analytically (10) and clinically validated across multiple independent studies and trials (8, 9, 11–19), two patient-level meta-analyses (8, 9), and are supported by high levels of evidence (7). Although prior studies explored limited associations between HER2DX scores and histopathologic features (20), a comprehensive, directly comparative analysis of these relationships has, to the best of our knowledge, not been reported.

This study aimed to systematically evaluate the correlation between HER2DX scores and conventional pathologic variables in a large, prospectively collected, real-world cohort. To the best of our knowledge, this is the first report to examine the concordance and complementarity between HER2DX genomic outputs and standard histopathology.

## Materials and Methods

### Study design and patient cohort

This was a multicenter, observational study that included tumor samples from 410 patients with newly diagnosed, stage I to III HER2+ breast cancer. Patients were diagnosed between January 2022 and June 2025 at 24 hospitals across Spain. Eligibility criteria included stage I to III HER2+ disease, availability of formalin-fixed, paraffin-embedded tumor tissue, and documented patient consent for genomic testing and translational research. Tumor material consisted primarily of core biopsy specimens (74%), with a smaller proportion of surgical specimens (26%). This distribution reflects the intended clinical use of HER2DX, which is typically performed on biopsy material to guide first treatment decisions.

HER2 positivity was determined locally at each participating site according to the corresponding 2023 American Society of Clinical Oncology/College of American Pathologists (ASCO/CAP) guidelines (4), based on IHC and/or *in situ* hybridization (ISH). Hormone receptor (HR) status (21) and Ki67 proliferation index (22) were also assessed locally by standard IHC methods.

### HER2DX genomic testing

All tumor samples underwent centralized HER2DX testing using a standardized platform (Reveal Genomics; ref. 10). All testing was performed centrally and blinded to pathologic and clinical outcomes. HER2DX provides three independent genomic scores (7). Tumor and nodal stage were provided by the treating physicians at the time of test ordering. When surgery had not yet been performed, staging was determined by standard clinical procedures; when surgery had been performed, staging was obtained from the surgical pathology report.

The HER2DX relapse risk score is a continuous variable ranging from 1 to 99 that integrates gene expression data and clinical features (i.e., tumor and nodal stages) to estimate the likelihood of distant recurrence. This score was developed using samples from the SHORT-HER randomized trial (23) and has been validated in more than 2,500 patients across multiple studies (9). The predefined cutoff separates tumors into low- and high-risk groups, with the low-risk category corresponding to a ≥90% probability of recurrence-free survival at 3, 5, and 7 years (6).

The HER2DX pCR likelihood score also ranges from 1 to 99 and estimates the probability of achieving a pCR after neoadjuvant therapy (6). It incorporates both molecular and clinical variables, including tumor size and nodal status. Tertile-based cutoffs (33 and 68) are used to classify tumors into pCR-low (1–32), pCR-medium (33–67), and pCR-high (68–99) categories. This score has shown strong predictive value in more than 700 patients across various neoadjuvant regimens, independent of HR status (8).

The HER2DX *ERBB2* mRNA score provides a continuous measure of *ERBB2* gene expression and is reported on a scale from 1 to 99 (6). Two cutoffs are used to define ERBB2-low (1–32), ERBB2-medium (33–50), and ERBB2-high (51–99) categories. The first cutoff aligns with the clinical threshold distinguishing HER2-negative and HER2+ disease, whereas the second defines the upper third of HER2+ tumors in terms of *ERBB2* expression. Because of potential interlaboratory variability in pathology assessments, ERBB2-low cases are recommended to be reevaluated by IHC/ISH to confirm HER2 status.

### Central pathologic review

Pathology review was conducted centrally by expert pathologists to standardize evaluation across centers. Stromal tumor-infiltrating lymphocytes (TIL) were quantified as a percentage of the stromal area occupied by mononuclear inflammatory cells, following International Immuno-Oncology Biomarker Working Group guidelines (24). The presence or absence of tertiary lymphoid structures (TLS) was assessed based on hematoxylin and eosin staining. Spatial patterns of the tumor-immune microenvironment (TIME) were categorized as immune-inflamed (dense infiltration of immune cells within tumor nests), immune-excluded (peripheral or stromal infiltration of immune cells without intratumoral penetration), or immune-desert (minimal or absent lymphocytic infiltration). Finally, histologic subtype was determined according to standard World Health Organization criteria (25).

### Statistical analysis

Descriptive statistics were used to summarize patient and tumor characteristics across the cohort. Continuous variables were reported as medians with ranges, and categorical variables as counts and percentages. Comparisons between HER2DX scores (pCR, relapse risk, and *ERBB2* mRNA expression scores) and clinicopathologic features were conducted using Student *t* test or one-way ANOVA for normally distributed continuous variables and Mann–Whitney U or Kruskal–Wallis tests when distributions were non-normal. *χ*^2^ or Fisher exact tests were used for categorical comparisons.

Pearson’s correlation coefficients were computed to evaluate associations between continuous HER2DX scores (including IGG, proliferation, and luminal gene signatures) and pathologic features such as Ki67 and stromal TIL. Heatmaps were used to visualize correlation patterns among HER2DX genomic signatures and pathologic variables.

For analyses of pCR, patients with available clinical and surgical outcome data after neoadjuvant trastuzumab-based therapy were included. pCR was defined as the absence of residual invasive disease in the breast and axillary lymph nodes (ypT0/is ypN0). Univariate and multivariable logistic regression models were used to evaluate associations between clinicopathologic variables, HER2DX pCR score, and pCR. OR with 95% confidence intervals (CI) were estimated, and variables with *P* < 0.10 in the univariable analysis were considered for inclusion in the multivariable model. Statistical significance was set at a two-sided *P* < 0.05. All statistical analyses and plots were performed using R (v4.3.2).

### Ethical considerations

All procedures involving human participants were conducted in accordance with recognized ethical standards, including the Declaration of Helsinki, the International Ethical Guidelines for Biomedical Research Involving Human Subjects, the Belmont Report, and the US Common Rule. The study protocol and the use of clinical and molecular data were reviewed and approved by the Institutional Review Board of Hospital Clínic Barcelona. Written informed consent for participation and for the use of clinical and genomic data for research purposes was obtained from all individuals before inclusion. HER2DX testing was performed as part of routine clinical care. Data handling, storage, and analysis complied with all applicable data protection regulations, including the European General Data Protection Regulation.

## Results

### Patient and tumor characteristics

**Figure 1.**
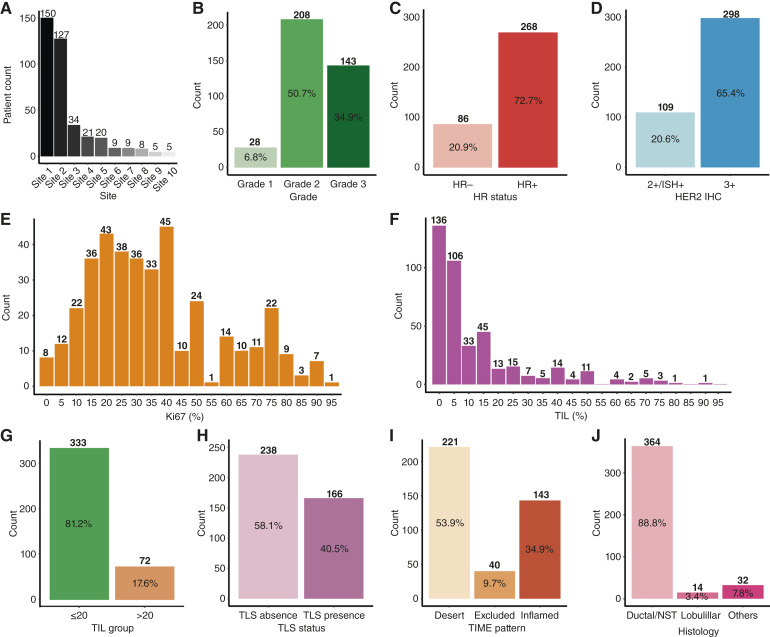
Overview of patient and tumor characteristics. Patient distribution across the top 10 hospitals by enrollment (**A**), tumor grade (**B**), HR status (**C**), HER2 IHC status (**D**), Ki67 expression (**E**), stromal TIL percentage (**F**), TIL grouped as <20% vs. ≥20% (**G**), TLS status (**H**), spatial immune distribution (**I**), and histologic subtype (**J**). Each panel summarizes the distribution of clinical and pathologic variables across the full study cohort (*N* = 410).

A total of 410 patients with stage I to III HER2+ breast cancer were included from 24 institutions across Spain. The distribution of clinical and pathologic features is shown in Fig. 1. The most frequent tumor grade was grade 2 (50.7%), 72.7% of tumors were HR-positive, 65.4% had HER2 3+ IHC status, and the median Ki67 and stromal TIL were 30% (range, <1–95%) and 6% (range, <1–90%), respectively. TLS were present in 40.5% of tumors, and the most common TIME spatial distribution was immune-desert (53.9%). Most tumors (88.8%) were histologically classified as invasive breast carcinoma of no special type (NST).

### Tumor and nodal stage associations

Tumor stage was significantly associated with three pathologic features (Supplementary Table S1). Compared with T1 to T2 tumors, T3 to T4 tumors were more frequently HER2 3+ (85.5% vs. 71.1%, *P* = 0.032) and exhibited higher proliferation (median Ki67, 40% vs. 30%; *P* = 0.029). In contrast, TLS were less frequently observed in T3 to T4 tumors than in T1 to T2 tumors (34.8% vs. 43.7%, *P* = 0.016). No significant associations were identified between tumor stage and histologic grade, HR status, or stromal TIL levels.

Nodal stage was associated with only one pathologic variable. Node-positive (N1–3) tumors displayed higher proliferation compared with node-negative (N0) tumors (median Ki67, 37% vs. 30%; *P* = 0.004). No significant differences were found for tumor grade, HER2 status, HR expression, TIL, TLS, immune spatial patterns, or histologic subtype.

### HER2DX pCR score associations

**Figure 2.**
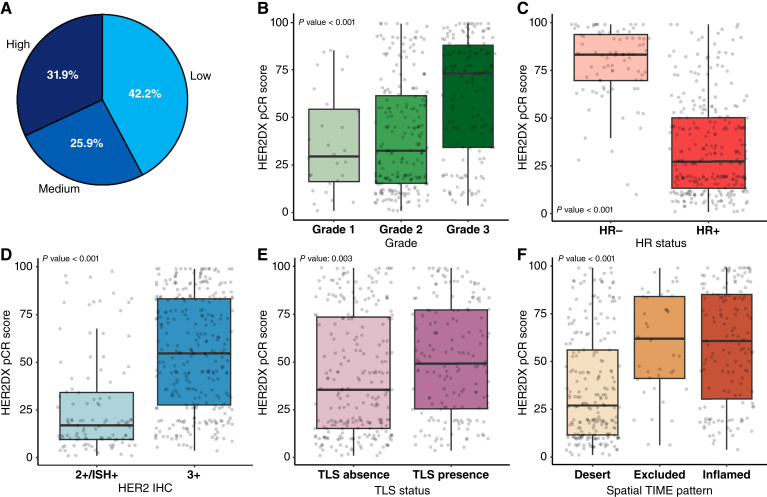
Associations between HER2DX pCR score and standard pathologic features. Distribution of HER2DX pCR score groups (low, medium, and high; **A**); HER2DX pCR score by tumor grade (**B**), HR status (**C**), HER2 IHC status (**D**), TLS status (**E**), and spatial immune distribution (**F**). All scores are shown on a continuous scale from 1 to 99. Boxplots show IQR with medians indicated and display score distributions across groups. Statistical comparisons were performed using appropriate parametric tests.

The HER2DX pCR likelihood score classified 42.2% of tumors as low, 25.9% as medium, and 31.9% as high (Fig. 2A). This score showed statistically significant associations with all pathology and immune features evaluated (Table 1; Fig. 2B–F). Tumors with high pCR scores were more likely to be grade 3 (55.2% in the high group vs. 23.8% in the low group, *P* < 0.001), HER2 3+ (90.1% vs. 51.4%, *P* < 0.001), and HR-negative (75.6% vs. 4.6%, *P* < 0.001). Additionally, higher pCR scores were significantly associated with increased proliferation (median Ki67, 40% vs. 25%; *P* < 0.001) and greater immune activation, as reflected by elevated TIL (median TIL: 15% vs. 4%, *P* < 0.001), presence of TLS (TLS, 42.7% vs. 33%; *P* = 0.003), and inflamed or immune-excluded spatial immune patterns (64.1% in the high group vs. 27.2% in the low group, *P* < 0.001). Finally, histologic subtype was also significantly associated with the pCR score (*P* = 0.023), with invasive breast carcinoma of NST being more frequent in the high-score group (31.9%) and lobular histology enriched in the low-score group (71.4%).

**Table 1.** Associations between HER2DX scores and histopathologic variables.

	HER2DX pCR likelihood score					HER2DX relapse risk score				HER2DX *ERBB2* mRNA score				
	Low	Medium	High	*P* value group distribution	*P* value continuous variable	Low	High	*P* value group distribution	*P* value continuous variable	Low	Medium	High	*P* value group distribution	*P* value continuous variable
	(*n* = 173)	(*n* = 106)	(*n* = 131)			(*n* = 224)	(*n* = 186)			(*n* = 57)	(*n* = 92)	(*n* = 261)		
Grade 1	16 (57.1%)	7 (25%)	5 (17.9%)	**<0.001**	**<0.001**	18 (64.3%)	10 (35.7%)	0.548	0.171	3 (10.7%)	6 (21.4%)	19 (67.9%)	0.666	0.609
Grade 2	105 (50.5%)	62 (29.8%)	41 (19.7%)			116 (55.8%)	92 (44.2%)			29 (13.9%)	53 (25.5%)	126 (60.6%)		
Grade 3	34 (23.8%)	30 (21%)	79 (55.2%)			76 (53.1%)	67 (46.9%)			20 (14%)	27 (18.9%)	96 (67.1%)		
2+/ISH+	81 (74.3%)	15 (13.8%)	13 (11.9%)	**<0.001**	**<0.001**	67 (61.5%)	42 (38.5%)	0.127	**0.009**	53 (48.6%)	36 (33.1%)	20 (18.3%)	**<0.001**	**<0.001**
3+	89 (29.9%)	91 (30.5%)	118 (39.6%)			156 (52.3%)	142 (47.7%)			4 (1.3%)	55 (18.5%)	239 (80.2%)		
HR−	4 (4.6%)	17 (19.8%)	65 (75.6%)	**<0.001**	**<0.001**	49 (57%)	37 (43%)	1.000	0.455	6 (7%)	14 (16.3%)	66 (76.7%)	**0.007**	**<0.001**
HR+	154 (57.5%)	77 (28.7%)	37 (13.8%)			153 (57.1%)	115 (42.9%)			45 (16.8%)	66 (24.6%)	157 (58.6%)		
Ki67 (%)	25 (<1 to 90)	32.5 (3–95)	40 (8–90)	**<0.001**	**<0.001**	28 (<1 to 90)	37 (<1 to 95)	**<0.001**	**<0.001**	30 (1–95)	30 (<1 to 90)	35 (2–90)	0.131	0.525
median (range)														
TIL (%)	4 (<1 to 50)	8 (<1 to 75)	15 (<1 to 90)	**<0.001**	**<0.001**	8 (<1 to 90)	5 (<1 to 70)	**0.009**	**<0.001**	5 (<1 to 50)	5 (<1 to 75)	8 (<1 to 90)	0.336	0.226
median (range)														
TLS absence	116 (48.7%)	52 (21.9%)	70 (29.4%)	**0.010**	**0.003**	122 (51.3%)	116 (48.7%)	0.118	**0.003**	38 (16%)	53 (22.2%)	147 (61.8%)	0.452	0.499
TLS presence	57 (34.3%)	53 (32%)	56 (33.7%)			99 (59.6%)	67 (40.4%)			19 (11.4%)	39 (23.5%)	108 (65.1%)		
Immune-desert	129 (58.4%)	50 (22.6%)	42 (19%)	**<0.001**	**<0.001**	116 (52.5%)	105 (47.5%)	0.352	**<0.001**	35 (15.8%)	55 (24.9%)	131 (59.3%)	0.299	0.531
Immune-excluded	6 (15%)	15 (37.5%)	19 (47.5%)			20 (50%)	20 (50%)			7 (17.5%)	6 (15%)	27 (67.5%)		
Immune-inflamed	38 (26.6%)	40 (28%)	65 (45.4%)			85 (59.4%)	58 (40.6%)			15 (10.5%)	31 (21.7%)	97 (67.8%)		
Ductal/NST	151 (41.5%)	97 (26.6%)	116 (31.9%)	0.093	**0.023**	199 (54.7%)	165 (45.3%)	0.306	0.585	52 (14.3%)	80 (22%)	232 (63.7%)	0.521	0.290
Lobulillar	10 (71.4%)	3 (21.4%)	1 (7.2%)			10 (71.4%)	4 (28.6%)			3 (21.4%)	4 (28.6%)	7 (50%)		
Others	12 (37.5%)	6 (18.7%)	14 (43.8%)			15 (46.9%)	17 (53.1%)			2 (6.3%)	8 (25%)	22 (68.7%)		

Summary of statistical associations between the three primary HER2DX genomic scores—pCR score (continuous), relapse risk score (continuous), and *ERBB2* mRNA expression score (continuous)—and eight key histopathologic features: HR status, tumor grade, Ki67 index, HER2 IHC score, stromal TIL, presence of TLS, spatial TIL distribution, and histologic subtype (ductal or invasive breast carcinoma of NST, lobular or others). Statistical significance was assessed using *t* tests or one-way ANOVA for continuous variables and *χ*^2^ test for categorical variables. Significant associations (*P* < 0.05) are highlighted in bold.

To evaluate whether HR status could confound the observed associations of the HER2DX pCR score, we performed bivariate logistic regression models including HR status as a covariate. The results confirmed that the main associations remained significant after adjustment for HR status (Supplementary Table S2). Next, separate models were fitted for the HER2DX pCR score (high vs. medium/low), including all pathologic features that were significant in univariate comparisons. In this model, higher grade, HR negativity, and HER2 IHC 3+ status were each independently associated with increased odds of being classified as HER2DX pCR–high (all *P* < 0.05; Supplementary Table S3).

### HER2DX relapse risk score associations

**Figure 3.**
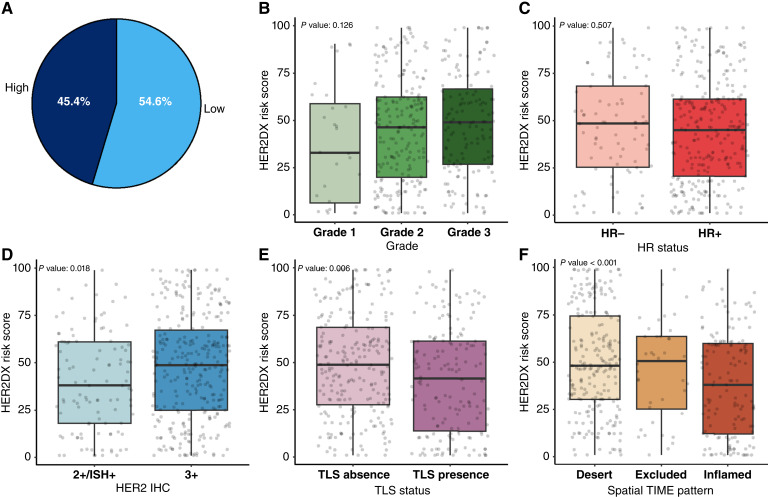
Associations between HER2DX risk score and pathologic variables. Distribution of HER2DX risk groups (low vs. high; **A**); HER2DX risk score by tumor grade (**B**), HR status (**C**), HER2 IHC status (**D**), TLS status (**E**), and spatial immune distribution (**F**). All scores are shown on a continuous scale from 1 to 99. Boxplots show IQR with medians indicated and display score distributions across groups. Statistical comparisons were performed using appropriate parametric tests.

Among all tumors, 54.6% were classified as low-risk tumors and 45.4% as high-risk tumors according to the HER2DX relapse risk score (Fig. 3A). This score showed statistically significant associations with several key histopathologic and immune-related features (Fig. 3; Table 1). Tumors with high relapse risk scores were more likely to exhibit elevated proliferation rates, as indicated by higher Ki67 values (*P* < 0.001), and were also characterized by reduced immune infiltration. Specifically, higher scores were associated with lower TIL levels (*P* = 0.009), absence of TLS (*P* = 0.003), and a predominance of immune-desert spatial TIME patterns (*P* < 0.001). Additionally, a modest but statistically significant association was observed with HER2 IHC status, with 3+ tumors exhibiting slightly higher scores than 2+/ISH+ tumors (*P* = 0.018). In contrast, no significant differences in risk scores were observed according to tumor grade, HR status, or histologic subtype.

To explore potential confounding by HR status, we also ran bivariate logistic regression models that included this variable alongside the HER2DX risk score. After adjustment, the key associations remained statistically significant (Supplementary Table S2).

We then built separate models for the HER2DX relapse risk score (high vs. low), incorporating all pathologic features that showed significance in univariate analyses. In these models, elevated Ki67 and reduced TIL levels were each independently linked to higher odds of being classified as HER2DX high risk (all *P* < 0.05; Supplementary Table S3).

### HER2DX *ERBB2* mRNA score associations

**Figure 4.**
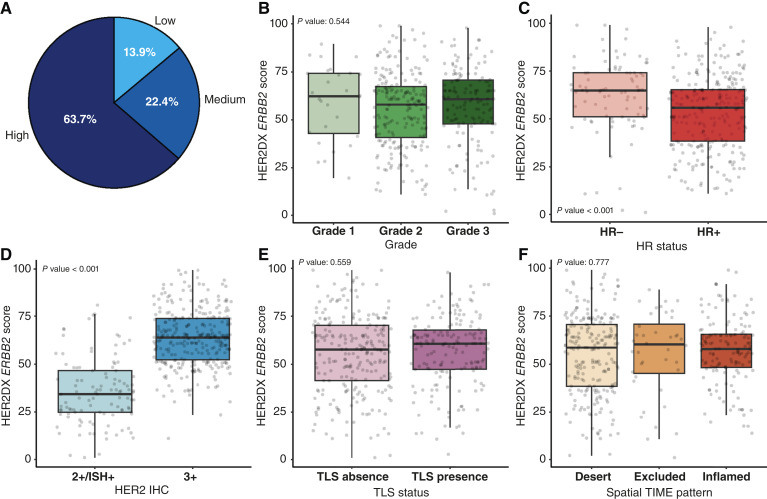
Associations between HER2DX *ERBB2* score and pathologic variables. Distribution of HER2DX *ERBB2* score groups (low, medium, and high; **A**); HER2DX *ERBB2* score by tumor grade (**B**), HR status (**C**), HER2 IHC status (**D**), TLS status (**E**), and spatial immune distribution (**F**). All scores are shown on a continuous scale from 1 to 99. Boxplots show IQR with medians indicated and display score distributions across groups. Statistical comparisons were performed using appropriate parametric tests.

The HER2DX *ERBB2* mRNA expression score classified 13.9% of tumors as low, 22.4% as medium, and 63.7% as high (Fig. 4A). This score showed strong associations with two key pathologic features: HER2 IHC and HR status (Table 1; Fig. 4B–F). *ERBB2* scores were significantly higher in HER2 3+ tumors compared with HER2 2+/ISH+ tumors (median, 63 vs. 39; *P* < 0.001) and in HR-negative compared with HR-positive tumors (median, 68 vs. 53; *P* < 0.001). Concordance between HER2 IHC categories and HER2DX *ERBB2* score groups was ∼81%, with a Cohen’s kappa coefficient of 0.56, indicating moderate agreement. Notably, ∼20% of HER2 3+ tumors were classified as low/medium by the *ERBB2* score, whereas ∼18% of HER2 2+/ISH+ tumors were classified as high. These findings reflect the biological link between *ERBB2* mRNA expression and HER2 protein overexpression and the known inverse relationship between *ERBB2* levels and HR signaling. In contrast, no statistically significant associations were observed between *ERBB2* mRNA scores and tumor grade, TLS status, immune spatial pattern, TIL percentage, Ki67, or histologic subtype.

To assess whether HR status influenced the observed relationships with the HER2DX *ERBB2* score, we performed bivariate logistic regression analyses including this factor. The results confirmed that the main associations remained significant after adjustment for HR status (Supplementary Table S2). Finally, separate models were fitted for the HER2DX *ERBB2* score (high vs. medium/low), including all pathologic features that were significant in univariate comparisons. In this model, only HER2 IHC 3+ was independently associated with higher odds of being classified as HER2DX ERBB2–high (*P* < 0.05; Supplementary Table S3).

Finally, among the 57 tumors classified as HER2DX ERBB2–low, 14 cases (24.6%) underwent central reevaluation by IHC and ISH according to ASCO/CAP guidelines. Of these, one case (7.2%) was reclassified as 0, two (14.3%) as 1+, five (35.7%) as 2+/ISH-negative, three (21.4%) as HER2+ (2+/ISH-amplified), and three (21.4%) showed intratumoral HER2 heterogeneity (Supplementary Fig. S1).

### HER2DX gene signature correlations with pathologic features

**Figure 5.**
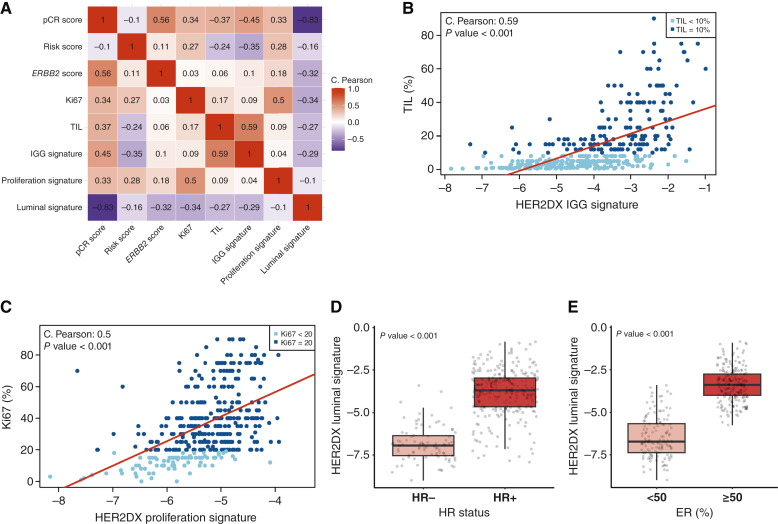
Correlations between HER2DX molecular modules and pathology features. Heatmap of Pearson correlation coefficients between HER2DX scores/modules (pCR score, risk score, *ERBB2* score, IGG, proliferation, and luminal) and pathologic features (TIL%, Ki67, HR status, TLS, HER2 IHC, tumor grade, and spatial pattern; **A**); scatter plots showing IGG score vs. TIL% (**B**), proliferation score vs. Ki67% (**C**), luminal score vs. binary HR status (**D**), and luminal score vs. ER % levels (**E**). Correlation values and *P* values are shown; positive correlations reflect biological concordance between genomic and pathologic features. Statistical comparisons were performed using appropriate parametric tests.

To further investigate the biological underpinnings of the HER2DX scores, we assessed Pearson correlations between HER2DX outputs (pCR, risk, and *ERBB2* scores), established gene expression signatures (IGG, proliferation, and luminal), and key pathologic variables (Fig. 5A–E). The IGG signature showed a positive correlation with TIL (r = 0.59, *P* < 0.001), consistent with its immunoglobulin-related composition, and was also positively associated with the HER2DX pCR score (r = 0.45, *P* < 0.001). The proliferation signature correlated most strongly with Ki67 (r = 0.50, *P* < 0.001) and showed moderate positive correlations with the pCR score (r = 0.33, *P* < 0.001) and HER2DX risk score (r = 0.28, *P* < 0.001). The luminal signature was inversely correlated with the pCR score (r = −0.83, *P* < 0.001) and Ki67 (r = −0.34, *P* < 0.001), consistent with lower pCR rates in luminal-like tumors. It also showed a weak negative correlation with the *ERBB2* score (r = −0.32, *P* < 0.001) and TIL (r = −0.27, *P* < 0.001) and a moderate concordance with HR status (Cohen’s kappa coefficient = −0.44; Supplementary Table S4).

### HER2DX pCR score and pathologic variables’ associations with pCR

To further explore the determinants of pCR, we analyzed 250 of 410 (61%) patients with available clinical information who were treated with neoadjuvant trastuzumab-based therapy and had corresponding surgical outcome data. Overall, 90 (36%) patients achieved a pCR.

In the univariate analysis, several pathologic variables were significantly associated with pCR (Supplementary Table S5). Tumors that were grade 3 (OR, 1.85; 95% CI, 1.07–3.22; *P* = 0.029), HER2 3+ by IHC (OR, 3.39; 95% CI, 1.67–7.47; *P* = 0.001), and HR-negative (OR, 0.26; 95% CI, 0.13–0.51; *P* < 0.001) were more likely to achieve pCR. Higher Ki67 (OR, 1.02; 95% CI, 1.00–1.03; *P* = 0.006) and higher stromal TIL (OR, 1.02; 95% CI, 1.00–1.03; *P* = 0.031) were also significantly associated with pCR, whereas TLS presence, immune-inflamed spatial patterns, and histologic subtype were not. In the multivariable analysis, the HER2DX pCR score remained the only independent predictor of pCR (OR, 1.77; 95% CI, 1.08–2.97; *P* = 0.030).

## Discussion

This is the first study to comprehensively evaluate the association between HER2DX genomic scores and histopathologic variables in stage I to III HER2+ breast cancer. By integrating genomic data with standard pathology across 410 prospectively accrued cases, we show that HER2DX scores are not only biologically coherent but also capture unique information complementary to traditional pathology.

The HER2DX pCR score demonstrated alignment with known predictors of therapy response, including HR status (5), tumor grade (26), Ki67 (22), TIL levels (24), and the presence of TLS (27). These associations confirm the biological basis of HER2DX pCR score, which is derived from tumor-intrinsic and immune features (6). However, HER2DX adds value by integrating these features into a robust molecular model that is reproducible, standardized, and independent of interobserver variability (10). The clinical utility of the pCR score has been supported by several validation studies (6, 8, 12–14, 19), and our current results underscore its ability to quantify immune-, luminal-, cell cycle–, and HER2-driven breast cancer biology beyond classic pathology.

The HER2DX risk score, designed to predict distant recurrence-free survival, was less strongly associated with HER2 IHC or HR status, highlighting its independence from traditional clinical subgroups. Instead, it reflects immune, luminal, and proliferative axes of tumor biology, offering a more nuanced estimation of risk (6). The positive correlations observed between the relapse risk and pCR scores with Ki67 and the proliferation signature were expected. Proliferation reflects a dual biological role in HER2+ breast cancer, as highly proliferative tumors tend to be more sensitive to systemic therapy (higher pCR) but also carry a greater baseline risk of recurrence if residual disease remains (6). Importantly, the risk score has been validated in diverse cohorts and remains prognostic even in patients with low-risk stage I disease treated with less intense systemic therapy, such as in the ATEMPT (16) and APT (17) trials. Furthermore, in both the APT (17) and RESPECT (28) studies, neither TIL nor HR status were prognostic, whereas the HER2DX risk score and IGG signature consistently stratified long-term outcomes (17). Importantly, our findings also align with the recent results of HER2DX in the CompassHER2 pCR trial (EA1181, NCT04266249) across 569 tumors (29), in which the HER2DX pCR, HER2DX IGG, and *ERBB2* scores, as well as intrinsic subtype, were independently associated with pCR beyond what is captured by HR status and HER2 IHC levels (29). These findings support the utility of HER2DX as a clinically deployable tool that recapitulates multiple biologically relevant tumor features, such as proliferation, immune infiltration, and luminal differentiation, in a standardized genomic format, thereby providing information that is otherwise difficult to assess consistently through pathology alone.

The HER2DX *ERBB2* score showed a strong positive correlation with HER2 IHC staining but, as expected, captured less biological variability then the pCR and risk scores within the HER2+ category (6). The continuous HER2DX *ERBB2* score may help refine treatment decisions, particularly in cases with borderline HER2 expression or incomplete IHC/ISH concordance. Moreover, approximately 10% to 15% of HER2+ tumors are classified as ERBB2-low by HER2DX, a subset shown in prior studies to be less responsive to HER2-targeted therapies (30, 31). In fact, according to our results, 57.1% of HER2DX ERBB2–low tumors are not confirmed centrally as HER2+, and 21.4% showed the presence of HER2 heterogeneity. Overall, the HER2DX *ERBB2* mRNA score provides a focused measure of HER2 pathway activation that is relatively independent from immune or proliferative features.

Our results reinforce the relevance of the TIME in early-stage HER2+ breast cancer. Both stromal TIL and TLS presence were strongly associated with HER2DX pCR and risk scores, consistent with prior evidence showing that immune-rich tumors, known as hot tumors, achieve higher response rates to neoadjuvant HER2-targeted therapy and better outcomes (27, 32–34). TLS, ectopic nonencapsulated lymphoid organized aggregates of B and T cells, have recently emerged as predictive and prognostic markers (27, 32) and were prevalent in nearly 50% of our cohort. Their association with higher TIL, HER2DX immune score, and pCR likelihood supports their role as markers of effective antitumor immunity in HER2+ disease.

Moreover, our findings echo recent results demonstrating that high expression of the HER2DX 14-gene IGG signature across all breast cancer subtypes is associated with significantly improved long-term survival in breast cancer survivors, even in the absence of recurrence (20). This signature, which correlates with increased B-cell and T-cell receptor diversity and the presence of TLS, may reflect a durable antitumor immune state that promotes not only immediate treatment response but also prolonged immune surveillance and patient longevity (20). That study showed that TLS presence and IGG signature expression were not entirely concordant: 25% of TLS-negative tumors had high IGG scores, whereas 45% of TLS-positive tumors exhibited low or medium IGG expression (20). In line with this, we also observed only moderate correlations between other genomic signatures and their corresponding pathologic features (e.g., IGG with TIL and proliferation with Ki67), underscoring that they are related but not redundant. Discordant cases likely reflect biological heterogeneity, sampling differences, or interobserver variability in pathologic scoring and may also capture transcriptional activity not fully evident by morphology.

Clinically, the ideal population for considering less intensive chemotherapy is patients with stage I to III HER2+ disease who are identified by HER2DX as pCR-high and -low risk or as low risk regardless of pCR score. In our cohort, this favorable group represented 57% of tumors in both HR-negative and HR-positive disease, although the underlying biology differed markedly between subtypes. Among HR-negative tumors, favorable cases were predominantly double favorable (pCR-high and -low risk) and were associated with HER2 IHC 3+, grade 3, high TIL, TLS presence, and inflamed TIME, consistent with HER2-addicted, immune-enriched tumors that are both chemosensitive and associated with excellent prognosis. In contrast, in HR-positive tumors, favorable cases were mainly low risk only, linking favorable outcomes to immune-luminal biology despite lower chemosensitivity and anti-HER2 sensitivity. These findings demonstrate that HER2DX can identify patients with favorable prognosis across HR subtypes, while clarifying the distinct pathologic and biological correlates underlying this classification.

A notable finding of our study is that approximately 10% to 15% of HER2+ tumors were classified as ERBB2-low by HER2DX, despite central IHC/ISH confirmation. This discordance highlights biological heterogeneity within the HER2+ category. Evidence from the metastatic setting consistently supports the clinical distinctiveness of this subgroup. In three independent cohorts—94 patients treated with first-line THP (31), 87 patients treated with T-DM1 (30), and 214 patients treated with first-line THP in the CLEOPATRA phase III trial (35)—HER2DX ERBB2–low tumors had inferior survival outcomes compared with ERBB2-high tumors, regardless of HER2 IHC status. These convergent results suggest that ERBB2-low tumors are less dependent on HER2 signaling and may derive limited benefit from standard HER2-targeted therapies. Although these data come from advanced disease, they raise important questions about the biology and management of ERBB2-low tumors in the early-stage setting.

Our study has some limitations. First, although pathology was centrally assessed for key variables such as TIL, TLS, TIME, and histologic subtype, HER2 IHC was performed locally at the participating institutions. As documented in prior studies, discordance between HER2 IHC and *ERBB2* gene expression is expected, even when central review is performed, because these methods capture different aspects of HER2 biology (protein vs. mRNA and categorical vs. continuous). Second, although tumor heterogeneity may also contribute to the observed variability, our dataset does not include multiregion or single-cell profiling to directly explore this. Future studies incorporating spatial and multi-sample profiling approaches will be important to further address this point. Despite these limitations, the associations observed were robust across univariate and multivariable analyses, underscoring the biological relevance of the HER2DX scores.

It is well-documented that standard pathology variables such as estrogen receptor (ER), progesterone receptor, Ki67, HER2, and histologic grade are subject to considerable interobserver and interlaboratory variability, even under central review (36). For example, standardization of Ki67 has proven extremely challenging across laboratories, ER levels assessed by IHC vary depending on methodology and cutoffs, and even tumor grade has shown discordance rates of up to 20% to 30% (36). Similar limitations apply to immune-related markers: TIL assessment, despite international guidelines (24), continues to show variability across pathologists and, importantly, lacks an established cutoff for clinical decision-making (33). For TLS, there is currently no validated or standardized scoring method in breast cancer. These limitations underscore why pathology, although essential for diagnosis, cannot always provide consistent and reproducible measures of tumor biology in large multicenter settings. In this context, HER2DX offers a standardized genomic platform that recapitulates multiple biological features in a reproducible manner, thereby complementing pathology and enhancing its clinical utility.

To conclude, HER2DX provides a reliable genomic tool that translates complex tumor biology into standardized scores, complements pathology, and supports personalized decision-making in HER2+ breast cancer.

## Supplementary Material

Supplementary Data 1 All the supplemental tables and figures

## Data Availability

All data generated or analyzed during this study are available from the corresponding author (A. Prat) upon reasonable request.
