# Supplementary material for: Associations of the HER2DX Genomic Test with Biological and Pathologic Features in HER2-Positive Breast Cancer
Source: Clin Cancer Res. 2025 Dec 1;32(3):570–80. doi: 10.1158/1078-0432.CCR-25-3123 (PMC12869160; doi:10.1158/1078-0432.CCR-25-3123)
Supplement: Supplementary Data 1 — All the supplemental tables and figures [file ccr-25-3123_supplementary_data_1_suppsd_1.pdf]

Supplemental Material

Associations of the HER2DX Genomic Test with Biological and Pathological Features in HER2-positive Breast Cancer

Supplementary Figure 1. HER2 IHC status in HER2DX ERRB2-low tumors. Representative images of HER2 IHC status (A); scheme of HER2DX *ERBB2*-low tumors repeated IHC results (B).

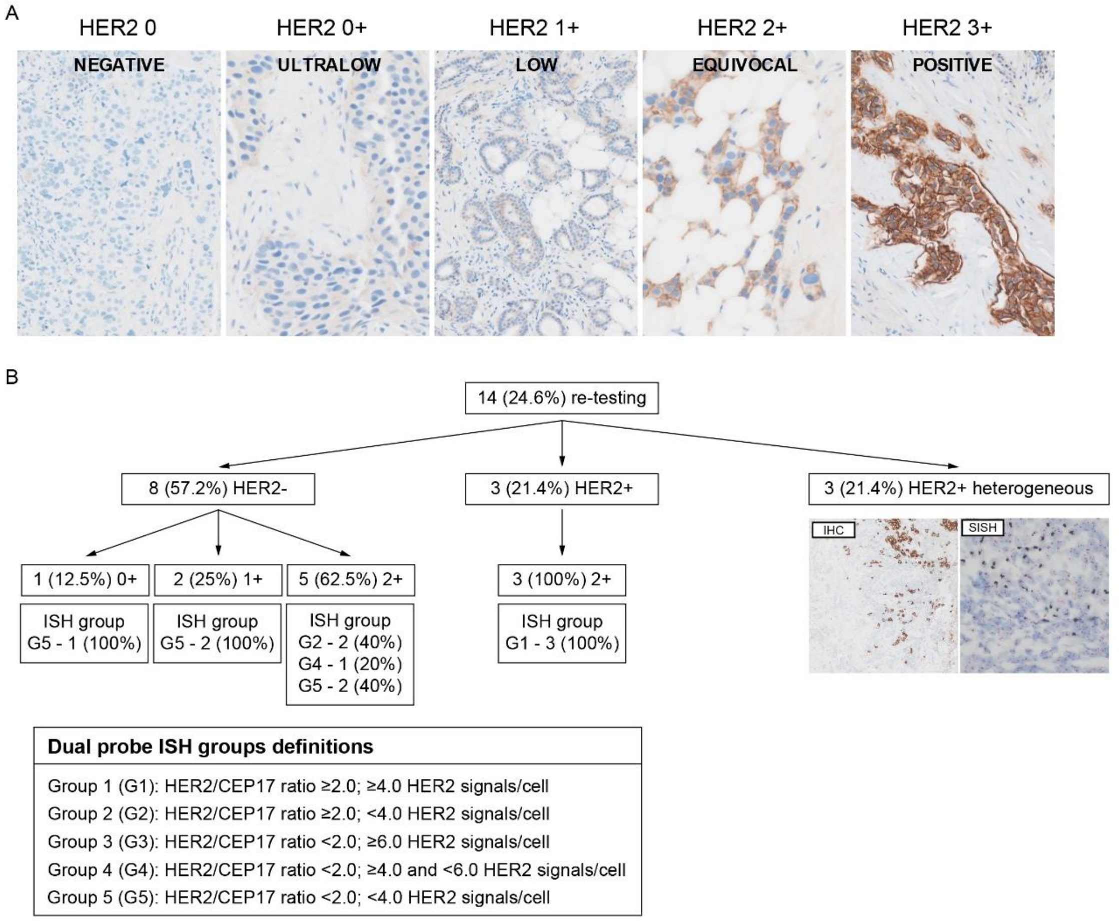

Supplementary Table 1. Distribution of pathological and immune features according to tumor and nodal stage.

	Tumor stage			Nodal stage		
	T1-2 (n = 348)	T3-4 (n = 62)	P-value	N0 (n = 255)	N1-3 (n = 154)	P-value
**Grade 1**	25 (89.3%)	3 (10.7%)	0.562	19 (67.9%)	9 (32.1%)	0.502
**Grade 2**	181 (87%)	27 (13%)		133 (63.9%)	75 (36.1%)	
**Grade 3**	119 (83.2%)	24 (16.8%)		84 (58.7%)	59 (41.3%)	
**2+/ISH+**	100 (91.7%)	9 (8.3%)	**0.032**	73 (67%)	36 (33%)	0.301
**3+**	246 (82.5%)	52 (17.5%)		181 (60.7%)	117 (39.3%)	
**HR-**	70 (81.4%)	16 (18.6%)	0.412	55 (64%)	31 (36%)	1
**HR+**	230 (85.8%)	38 (14.2%)		173 (64.5%)	95 (35.5%)	
**Ki67 (%)** median (range)	30 (<1-95)	40 (<1-90)	**0.029**	30 (<1-95)	37 (<1-90)	**0.004**
**TILs (%)** median (range)	8 (<1-80)	5 (<1-90)	0.685	6 (<1-90)	8 (<1-75)	0.716
**TLS absence**	193 (86.6%)	30 (13.4%)	**0.016**	154 (64.7%)	84 (35.3%)	0.240
**TLS presence**	150 (90.4%)	16 (9.6%)		97 (58.4%)	69 (41.6%)	
**Immune-desert**	182 (82%)	40 (18%)	0.225	138 (62.4%)	83 (37.6%)	0.984
**Immune- excluded**	37 (92.5%)	3 (7.5%)		25 (62.5%)	15 (37.5%)	
**Immune-inflamed**	124 (86.7%)	19 (13.3%)		88 (61.5%)	55 (38.5%)	
**Ductal/NST**	311 (85.4%)	53 (14.6%)	0.471	226 (62.1%)	138 (37.9%)	0.377
**Lobulillar**	12 (85.7%)	2 (14.3%)		11 (78.6%)	3 (21.4%)	
**Others**	25 (78.1%)	7 (21.9%)		18 (56.2%)	14 (43.8%)	

Supplementary Table 2. Univariate and bivariate models adjusted by hormone receptor status for variables associated with HER2DX scores.

		Univariate analysis			Bivariate, adjusted by HR status		
	Variable	OR	95% CI	p-value	OR	95% CI	p-value
HER2DX pCR score	Grade 3 vs Grade 1/2	1.024	1.016-1.032	**< 0.001**	1.023	1.013-1.033	**< 0.001**
	HER2 3+ vs HER2 2+/ISH+	1.028	1.028-1.049	**< 0.001**	1.041	1.028-1.055	**< 0.001**
	Ki67 ≥20% vs Ki67 <20%	1.020	1.011-1.029	**< 0.001**	1.026	1.013-1.039	**< 0.001**
	TILs ≥30% vs TILs <30%	1.030	1.019-1.041	**< 0.001**	1.031	1.018-1.046	**< 0.001**
	TLS presence vs TLs absence	1.009	1.002-1.015	**0.009**	1.016	1.007-1.025	**< 0.001**
	Immune-inflamed vs Rest	1.021	1.014-1.028	**< 0.001**	1.026	1.016-1.036	**< 0.001**
	Ductal vs Rest	1.004	0.994-1.014	0.431	1.012	0.996-1.029	0.156
HER2DX risk score	Grade 3 vs Grade 1/2	1.006	0.999-1.014	0.115	1.008	0.999-1.016	0.074
	HER2 3+ vs HER2 2+/ISH+	1.010	1.002-1.019	**0.013**	1.009	1.000-1.018	0.058
	Ki67 ≥20% vs Ki67 <20%	1.023	1.013-1.034	**< 0.001**	1.022	1.012-1.034	**< 0.001**
	TILs ≥30% vs TILs <30%	0.975	0.963-0.986	**< 0.001**	0.974	0.961-0.986	**< 0.001**
	TLS presence vs TLs absence	0.990	0.982-0.997	**0.005**	0.989	0.981-0.996	**0.005**
	Immune-inflamed vs Rest	0.985	0.977-0.993	**< 0.001**	0.986	0.978-0.994	**0.001**
	Ductal vs Rest	0.998	0.987-1.009	0.700	0.997	0.984-1.010	0.410
HER2DX ERBB2 score	Grade 3 vs Grade 1/2	1.003	0.993-1.014	0.558	1.000	0.988-1.012	0.996
	HER2 3+ vs HER2 2+/ISH+	1.114	1.091-1.140	**< 0.001**	1.103	1.081-1.129	**< 0.001**
	Ki67 ≥20% vs Ki67 <20%	1.016	1.004-1.029	**0.012**	1.015	1.002-1.029	**0.025**
	TILs ≥30% vs TILs <30%	1.007	0.993-1.022	0.338	1.002	0.986-1.019	0.802
	TLS presence vs TLs absence	1.003	0.993-1.013	0.597	1.007	0.996-1.019	0.225
	Immune-inflamed vs Rest	1.006	0.996-1.017	0.266	1.006	0.994-1.017	0.349
	Ductal vs Rest	0.996	0.980-1.011	0.598	0.997	0.978-1.016	0.745

Supplementary Table 3. Univariate and multivariable logistic regression models of factors associated with HER2DX scores

		Univariate analysis			Multivariable analysis		
	Variable	OR	95% CI	p-value	OR	95% CI	p-value
HER2DX pCR score	Grade 3 vs Grade 1/2	5.10	3.23-8.14	**< 0.001**	3.93	1.95-8.14	**< 0.001**
	HER2 3+ vs HER2 2+/ISH+	4.84	2.68-9.42	**< 0.001**	4.06	1.79-10.16	**0.001**
	HR+ vs HR-	0.05	0.03-0.09	**< 0.001**	0.07	0.03-0.13	**< 0.001**
	Ki67	1.03	1.02-1.04	**< 0.001**	1.01	1.00-1.03	0.105
	TILs	1.04	1.03-1.05	**< 0.001**	1.01	1.00-1.03	0.137
	TLS presence vs TLs absence	1.22	0.80-1.87	0.400	NA	NA	NA
	Immune-inflamed vs Rest	2.73	1.77-4.24	**< 0.001**	NA	NA	NA
	Ductal vs Rest	0.97	0.51-1.91	0.900	NA	NA	NA
HER2DX risk score	Grade 3 vs Grade 1/2	1.16	0.76-1.76	0.490	NA	NA	NA
	HER2 3+ vs HER2 2+/ISH+	1.45	0.93-2.28	0.100	NA	NA	NA
	HR+ vs HR-	1.00	0.61-1.663	0.990	NA	NA	NA
	Ki67	1.02	1.01-1.03	**< 0.001**	1.02	1.01-1.03	**< 0.001**
	TILs	0.98	0.97-1.00	**0.010**	0.98	0.96-0.99	**< 0.001**
	TLS presence vs TLs absence	0.71	0.48-1.06	0.100	NA	NA	NA
	Immune-inflamed vs Rest	0.74	0.49-1.12	0.160	NA	NA	NA
	Ductal vs Rest	0.99	0.53-1.84	0.970	NA	NA	NA
HER2DX ERBB2 score	Grade 3 vs Grade 1/2	1.28	0.83-1.99	0.265	NA	NA	NA
	HER2 3+ vs HER2 2+/ISH+	18.03	10.46-32.36	**< 0.001**	15.60	8.79-28.88	**< 0.001**
	HR+ vs HR-	0.43	0.24-0.74	**0.003**	0.67	0.34-1.25	0.200
	Ki67	1.01	1.00-1.02	0.158	NA	NA	NA
	TILs	1.01	0.99-1.02	0.308	NA	NA	NA
	TLS presence vs TLs absence	1.15	0.76-1.75	0.500	NA	NA	NA
	Immune-inflamed vs Rest	1.37	0.90-2.12	0.147	NA	NA	NA
	Ductal vs Rest	1.03	0.54-1.93	0.927	NA	NA	NA

Supplementary Table 4. Distribution of HER2DX luminal score groups according to HR IHC status.

HR IHC status	Luminal Low	Luminal Medium	Luminal High	Total
HR–	80 (93%)	4 (4.7%)	2 (2.3%)	86
HR+	34 (12.7%)	81 (30.2%)	153 (57.1%)	268
NA	36 (64.3%)	9 (16.1%)	11 (19.6%)	56
**Total**	150	94	166	410

Supplementary Table 5. Univariate and multivariable logistic regression models of factors associated with pCR.

	Univariate analysis			Multivariable analysis		
Variable	OR	95% CI	p-value	OR	95% CI	p-value
Grade 3 vs Grade 1/2	1.85	1.07-3.22	**0.029**	1.23	0.59-2.56	0.580
HER2 3+ vs HER2 2+/ISH+	3.39	1.67-7.47	**0.001**	2.77	1.12-7.50	**0.030**
HR+ vs HR-	0.26	0.13-0.51	**< 0.001**	0.68	0.28-1.63	0.380
Ki67	1.02	1.00-1.03	**0.006**	1.02	1.00-1.03	0.050
TILs	1.02	1.00-1.03	**0.031**	1	0.97-1.02	0.640
TLS presence vs TLs absence	1.03	0.61-1.74	0.915	NA	NA	NA
Immune-inflamed vs Rest	1.18	0.69-2.01	0.536	NA	NA	NA
Ductal/NST vs Rest	1.41	0.58-3.77	0.465	NA	NA	NA
HER2DX pCR score	2.25	1.65-3.14	**< 0.001**	1.77	1.08-2.97	**0.030**

Supplementary Table 6. Representativeness of Study Participants

Variables	Early-stage HER2-positive breast cancer (stage I–III)
Sex	HER2-positive breast cancer, like all breast cancer subtypes, predominantly affects women. Breast cancer in men represents <1% of all breast cancers, and HER2-positive disease in men is even rarer. Because the incidence of HER2-positive tumors is highest in women aged 40–60 years, most real-world cohorts and clinical trial populations consist almost exclusively of female patients.
Age	In population-level studies, the median age at diagnosis of HER2-positive early breast cancer ranges from 50 to 55 years, younger than the median age for all breast cancers (~62–63 years). The incidence is highest in premenopausal and perimenopausal women.
Race/ethnicity	Globally, HER2-positive breast cancer occurs across all racial/ethnic groups with modest variability. Slightly higher incidence is observed among Asian and Hispanic women. In Europe, race/ethnicity data are not routinely collected, but HER2-positive disease consistently represents 15–20% of invasive breast cancers.
Geography	HER2-positive breast cancer accounts for ~15–20% of invasive breast cancers worldwide. Incidence and mortality vary by region, primarily reflecting differences in access to early detection and anti-HER2 therapies.
Other considerations	HER2-positive tumors often show high proliferative activity and aggressive features. The advent of anti-HER2 therapies has significantly improved outcomes. pCR rates in neoadjuvant trials range from 40–60%, influenced by HR status and chemotherapy regimen.
Overall representativeness of this study	This study included patients with stage I–III HER2-positive breast cancer diagnosed and treated in routine clinical practice in Spain. Because HER2DX testing was ordered as part of standard care, the cohort reflects the population of individuals for whom clinicians requested genomic profiling during the study period, without additional eligibility restrictions related to age, sex, comorbidities, or tumor characteristics. Although demographic variables such as age and sex were not collected as part of this analysis, the population is expected to align with the typical distribution of early-stage HER2-positive disease seen in contemporary European practice. Overall, the study is considered representative of patients with early-stage HER2-positive breast cancer managed in real-world settings where HER2DX testing is implemented.
